# Phylogeny of Termites Inferred from Mitogenomic Data with a Large Taxon Sampling

**DOI:** 10.3390/genes17101310

**Published:** 2026-10-21

**Authors:** Nan Song, Junpeng Ji, Lijuan Su

**Affiliations:** 1 College of Plant Protection, Henan Agricultural University, Zhengzhou 450046, China; songnan@henau.edu.cn (N.S.); ad3041533192@126.com (J.J.); 2 College of Life Sciences, Henan Agricultural University, Zhengzhou 450046, China

**Keywords:** Termitoidae, Rhinotermitidae, mitogenome, phylogeny

## Abstract

**Background:** Termites (epifamily Termitoidae) represent the oldest known lineage of eusocial insects, yet their higher-level phylogenetic relationships remain contentious. **Methods:** We sequenced ten new termite mitochondrial genomes (mitogenomes) from Australia and China and integrated them with published data to assemble a comprehensive dataset of 560 species, including 553 termite ingroup species representing all 13 extant families and seven *Cryptocercus* outgroups. Phylogenetic relationships were inferred using maximum likelihood analyses of mitochondrial protein-coding genes and ribosomal RNA genes under amino acid and nucleotide datasets. **Results:** All newly sequenced mitogenomes contained the canonical set of 37 mitochondrial genes, exhibited a pronounced A+T bias, and retained the ancestral insect gene order. *Mastotermes darwiniensis* (Mastotermitidae) was consistently recovered as the sister lineage to all other termites. Among the basal lineages, the relationship ((Hodotermopsidae + Stolotermitidae) + (Archotermopsidae + Hodotermitidae)) received strong support. Kalotermitidae was recovered as monophyletic and as the sister group to Neoisoptera. The traditional Rhinotermitidae was non-monophyletic, and the analyses supported the revised family-level classification that restricts Rhinotermitidae to Rhinotermitinae while recognizing Psammotermitidae, Termitogetonidae, and Heterotermitidae as separate lineages. Within Termitidae, Macrotermitinae was recovered as the sister group to the remaining termitid lineages, whereas Sphaerotermitinae formed a distinct lineage, consistent with its recognition as a separate subfamily. **Conclusions:** Our results substantially expand mitogenomic sampling across termites and provide further support for recent family- and subfamily-level taxonomic revisions. The inferred relationships broadly agree with recent molecular studies, while some unresolved relationships require denser taxon sampling and nuclear genomic data.

## 1. Introduction

Termites (Insecta, Blattodea) represent the oldest known lineage of eusocial insects, comprising over 3000 described species worldwide [1]. Derived from within the wood-feeding cryptocercid roaches during the Late Jurassic (ca. 140–150 Ma), termites predated the origins of other major eusocial insects, such as ants and bees, by approximately 35 to 50 million years [2,3]. This independent transition to eusociality in hemimetabolous termites offers a striking example of convergent evolution with holometabolous Hymenoptera, separated by over 350 million years of divergence. Globally distributed between latitudes 45° N and 45° S [4,5], termite diversity is heavily concentrated in tropical regions [5,6]. The initial divergence of basal lineages predated the break-up of Gondwana [7]. While basal termites survived major biotic shifts in the Cretaceous with modest abundance, the Tertiary thermal maximum (late Paleocene to Eocene) and the widespread expansion of C4 grasslands in the Miocene triggered rapid evolutionary radiation [8]. This expansion is clearly reflected in the fossil record: termites’ fossils rose from <1% in the Cretaceous–early Tertiary to 5–10% in the Miocene, mainly due to Termitidae radiation [7]. Among extant taxa, the family Termitidae, which account for over 80% of all extant species, are dominant ecosystem engineers. In tropical and subtropical biomes, they serve as primary decomposers, consuming 50% to 100% of dead plant biomass and up to 90% of wood, litter, and soil organic matter in specific habitats [9,10]. By digesting the lignocellulose, the most abundant biomolecules on land, termites play a critical, unmatched role in global carbon cycling and soil nutrient dynamics [9,11,12,13].

Recent phylogenetic studies combining molecular and morphological data from extant species have significantly advanced our understanding of termite evolution [7,14,15,16,17,18,19,20,21,22,23,24,25,26,27,28]. While Kambhampati and Eggleton [29] reviewed early classification schemes, termite systematics has undergone substantial revision over the past century [7,19,30,31,32,33,34,35,36,37,38,39]. Historically, termites were divided into seven families (Mastotermitidae, Kalotermitidae, Hodotermitidae, Termopsidae, Rhinotermitidae, Serritermidae, and Termitidae) [40], but subsequent classifications expanded this to nine: Archotermopsidae, Mastotermitidae, Kalotermitidae, Hodotermitidae, Rhinotermitidae, Serritermitidae, Stolotermitidae, Stylotermitidae, and Termitidae [1]. Of these, the Archotermopsidae as a new family was established to include 3 extant genera of *Archotermopsis*, *Zootermopsis*, and *Hodotermopsis* [7]. However, subsequent studies indicated the paraphyly of Archotermopsidae relative to Hodotermitidae [15,17,22,25,41]. To resolve the paraphyly of Archotermopsidae relative to Hodotermitidae indicated by molecular phylogenies, the family Hodotermopsidae was later erected for *Hodotermopsis* [42,43]. Following the revised classification proposed by Hellemans et al. [19], the present study recognizes 13 extant termite families. These include Heterotermitidae, Psammotermitidae, and Termitogetonidae, which were formerly treated as subfamilies within Rhinotermitidae, while Rhinotermitidae *sensu stricto* is restricted to Rhinotermitinae.

Systematists generally divide termites into “lower termites” and the “higher termites” (Termitidae), with broad consensus that Mastotermitidae represents the basal lineage. However, some authors suggested that ‘Lower” and ‘‘higher” terms refer to a gradistic classification which is not really informative [44] and will not be used hereafter [25]. In addition, the phylogenetic placements of Kalotermitidae, Hodotermitidae, and Serritermidae varied across different studies [7,18,25,27,29,45,46]. Additionally, the exact phylogenetic relationships among the families of Neoisoptera and subfamilies of Termitidae remain the subject of ongoing debate [19]. The internal relationships remain contested for Rhinotermitidae. Rhinotermitidae is frequently recovered as non-monophyletic, and molecular evidence indicates that the rhinotermitid subfamily Heterotermitinae is paraphyletic with respect to Coptotermitinae. Thus, despite numerous robust phylogenetic analyses, deep family and subfamily relationships remain contentious.

Here, we sequenced and characterized ten novel termite mitochondrial genomes. By combining these data with previously published sequences, we assembled a comprehensive dataset comprising 560 terminal species, including 553 termite species that represent all 13 families and the major subfamilies of the largest two families, namely Rhinotermitidae, and Termitidae. Leveraging this broad mitogenomic sampling, we investigated phylogenetic relationships at the family and subfamily levels, aiming to provide new insights into the evolutionary history of termites.

## 2. Materials and Methods

### 2.1. Specimen Collection and Identification

Termite specimens representing ten species were collected from Australia and China between 2023 and 2025. *Coptotermes acinaciformis*, *Schedorhinotermes breinli* and *Tumuliterme* sp. AU_W3 were collected on the Sunshine Coast, Australia, between November and December 2023. Seven termite species were collected in Henan Province, China, for study: *Macrotermes barneyi*, *Odontotermes formosanus*, *Reticulitermes kanmonensis*, *R. qingjiangensis*, *R. citrinus*, and *R. leptomandibularis* were collected in Xinyang between 2024 and 2025, while *R. labralis* was collected in Sanmenxia in August 2023. Voucher numbers and detailed collection information are provided in Table S1. No specific permits were required for sampling. All specimens were preserved in absolute ethanol at −20 °C prior to DNA extraction.

Species identification was performed using an integrated approach combining adult morphology and molecular analysis. Morphological characters were examined using a Leica M205 A stereomicroscope (Leica Microsystems, Wetzlar, Germany) and identified using diagnostic characters described in the taxonomic literature [47,48,49,50], including the head capsule, pronotum, and mandible shape of soldiers, as well as body size and wing venation of reproductives. For molecular validation, mitochondrial *cox1* gene fragments were sequenced and queried against the Barcode of Life Data System (BOLD: https://www.boldsystems.org/index.php/IDS_OpenIdEngine, accessed on 1 August 2026) database [51]. Species designations were confirmed only for sequence matches yielding a sequence identity of ≥98%.

### 2.2. DNA Extraction and Genome Sequencing

Genomic DNA was extracted from the thoracic muscle tissue of an individual specimen preserved in 100% ethanol using the TIANamp Genomic DNA Kit (TIANGEN Biotech, Beijing, China) according to the manufacturer’s protocol. DNA concentration and purity were assessed using a Q5000 Micro-Volume UV-Vis Spectrophotometer (Quawell Technology Inc., San Jose, CA, USA).

Genomic DNA libraries with a target insert size of 400 bp were constructed using the TruSeq Nano DNA High Throughput Library Prep Kit (Illumina, Inc., San Diego, CA, USA) according to the manufacturer’s instructions. High-throughput sequencing was performed by Personal Biotechnology Co., Ltd. (Shanghai, China) on a DNBSEQ-T7RS platform using the DNBSEQ-T7RS High-Throughput Sequencing Set V3.0. Sequencing (MGI Tech Co., Ltd., Shenzhen, China) was conducted using a 150 bp paired-end sequencing, generating a minimum of 20 Gb of raw data per sample.

Raw read quality was initially assessed using Fastp (v0.23) [52]. Adapter sequences and low-quality bases were subsequently trimmed using Trimmomatic (v0.32) [53]. Only high-quality reads maintaining a Q30 score ≥ 96.21% were retained for downstream de novo assembly.

### 2.3. Mitogenome Assembly and Annotation

Mitogenomes were assembled de novo from filtered paired-end reads using GetOrganelle (v1.7.7.1) [54] with the “animal_mt” preset and GetOrganelleDB (v0.0.1) as the reference database. To complement these assemblies, MitoZ (v3.6) [55] was employed to identify and assemble target mitochondrial reads. Outputs from MitoZ were cross-checked against GetOrganelle assemblies to resolve potential sequence ambiguities and ensure complete mitogenome recovery. Filtered reads were remapped to the finalized assemblies using Geneious (R11) [56] to estimate nucleotide coverage depth.

Preliminary de novo annotation was performed using MITOS (v2) [57]. Boundary positions of protein-coding genes (PCGs) and the two ribosomal RNA (rRNA) genes were manually refined by aligning sequences against reference mitogenomes of closely related species retrieved from GenBank. Secondary structures of transfer RNA (tRNA) genes were predicted using MITOS (v2). Subsequent downstream analyses including gene extraction, nucleotide composition profiling, and gene order visualization were conducted using PhyloSuite (v2) [58,59]. The circular mitochondrial genome map was generated using Proksee (v2) [60], and the gene order map was produced with PhyloSuite (v2) [58,59]. The ten newly sequenced mitogenomes have been deposited in GenBank under accession numbers QB020495–QB020504.

### 2.4. Phylogenetic Analyses

Nucleotide sequences of the 13 protein-coding genes (PCGs) were aligned using MACSE (v2) [61], applying the invertebrate mitochondrial genetic code to preserve open reading frames and avoid frameshift disruptions. The poorly aligned regions and gaps were manually removed using MEGA (v12.1) [62]. Ribosomal RNA (rRNA) genes were aligned using MAFFT (v7) [63] with the E-INS-i strategy, followed by automated trimming using Gap Strip/Squeeze (v2.1.0) (http://www.hiv.lanl.gov/content/sequence/GAPSTREEZE/gap.html, accessed on 10 August 2026) at a 30% gap tolerance threshold to remove poorly aligned regions. Individual gene alignments were concatenated using PhyloSuite (v2) [58,59].

To evaluate phylogenetic signals and potential compositional biases, three distinct matrix datasets were compiled: (1) PCG_AA: Inferred amino acid sequences of the 13 PCGs; (2) PCG_NT123: Nucleotide sequences of the 13 PCGs, including all three codon positions; (3) PCG_rRNA: Combined nucleotide sequences of the 13 PCGs and two rRNA genes.

Species included in the phylogenetic analyses are listed in Table S1. We followed the family-level classification of Hellemans et al. [19] (Table S2). The dataset comprised 553 ingroup species representing 13 termite families [19] and seven outgroup species from the family Cryptocercidae. Maximum likelihood (ML) phylogenetic reconstructions were performed using IQ-TREE (v2.0) [64]. Datasets were partitioned by gene, and optimal substitution models were selected using ModelFinder [65] in IQTREE (Table S3). Node support was evaluated using 10,000 ultrafast bootstrap replicates [66].

To investigate potential compositional heterogeneity and substitution saturation in the mitogenomic datasets, we conducted additional analyses using AliGROOVE (v 1.06) [67] and DAMBE (v 7.3.11) [68]. Compositional heterogeneity was assessed using AliGROOVE for the amino acid dataset (PCG_AA). We further assessed the phylogenetic consequences of excluding the third codon position by reconstructing ML tree from the PCG_NT12 dataset, which included only the first and second codon positions. These additional analyses were used to assess the sensitivity of the inferred phylogenetic relationships to compositional heterogeneity, potential substitution saturation, and codon-position treatment.

## 3. Results

### 3.1. Genome Sequencing and Assembly

The total number of 150 bp paired-end reads ranged from 137,673,560 (*S. breinli*) to 228,100,670 (*R. labralis*), corresponding to minimum and maximum yields of 20.5 Gb (20,499,431,922 bp) and 33.5 Gb (33,528,772,832 bp), respectively. The ten newly sequenced mitogenomes ranged in length from 15,285 bp (*M. barneyi*) to 16,721 bp (*Tumulitermes* sp. AU_W3). Mean sequencing depth was consistently high across all samples. Specifically, *S. breinli*, *C. acinaciformis* and *Tumulitermes* sp. AU_W3 achieved mean depths of 16,812× (range: 1–33,855×), 19,291× (range: 75–36,914×) and 29,057× (range: 1–49,181×), respectively. Six additional species exceeded high coverage thresholds: *R. kanmonensis*, *R. qingjiangensis*, and *R. labralis* surpassed 4898× mean coverage (range: 1–12,823×), while *R. leptomandibularis*, *O. formosanus*, and *R. citrinus* exceeded 3432× mean coverage (range: 1–9856×). *M. barneyi* exhibited the lowest mean depth at 1357× (range: 1–4916×). These high sequencing depths indicate substantial mitochondrial read coverage, confirming high-confidence genome assemblies. Coverage plots for each mitogenome newly sequenced in this study are presented in Figure S1. While the vast majority of the newly sequenced mitogenomes exhibited high and relatively uniform coverage depths, localized drops down to a minimum coverage of 1× occasionally occurred, primarily at the terminal ends (Figure S1). These isolated dips typically stemmed from read-mapping edge effects at alignment boundaries or minor local variations across intergenic and low-complexity regions, without disrupting overall assembly continuity. The completeness, circularity, and structural integrity of each mitogenome were validated using the automated, graph-based workflow in GetOrganelle. Circularity was confirmed algorithmically through the assembly graph (FASTG/GFA format), where mitogenomes were designated as “circular” only when terminal repeats were successfully resolved into a single, continuous, closed-loop path, ensuring an artifact-free assembly. Uniformity in coverage depth and read-mapping consistency across the entire genome were further verified visually for all species (Figure S1).

### 3.2. Mitogenome Characteristics

**Figure 1:**
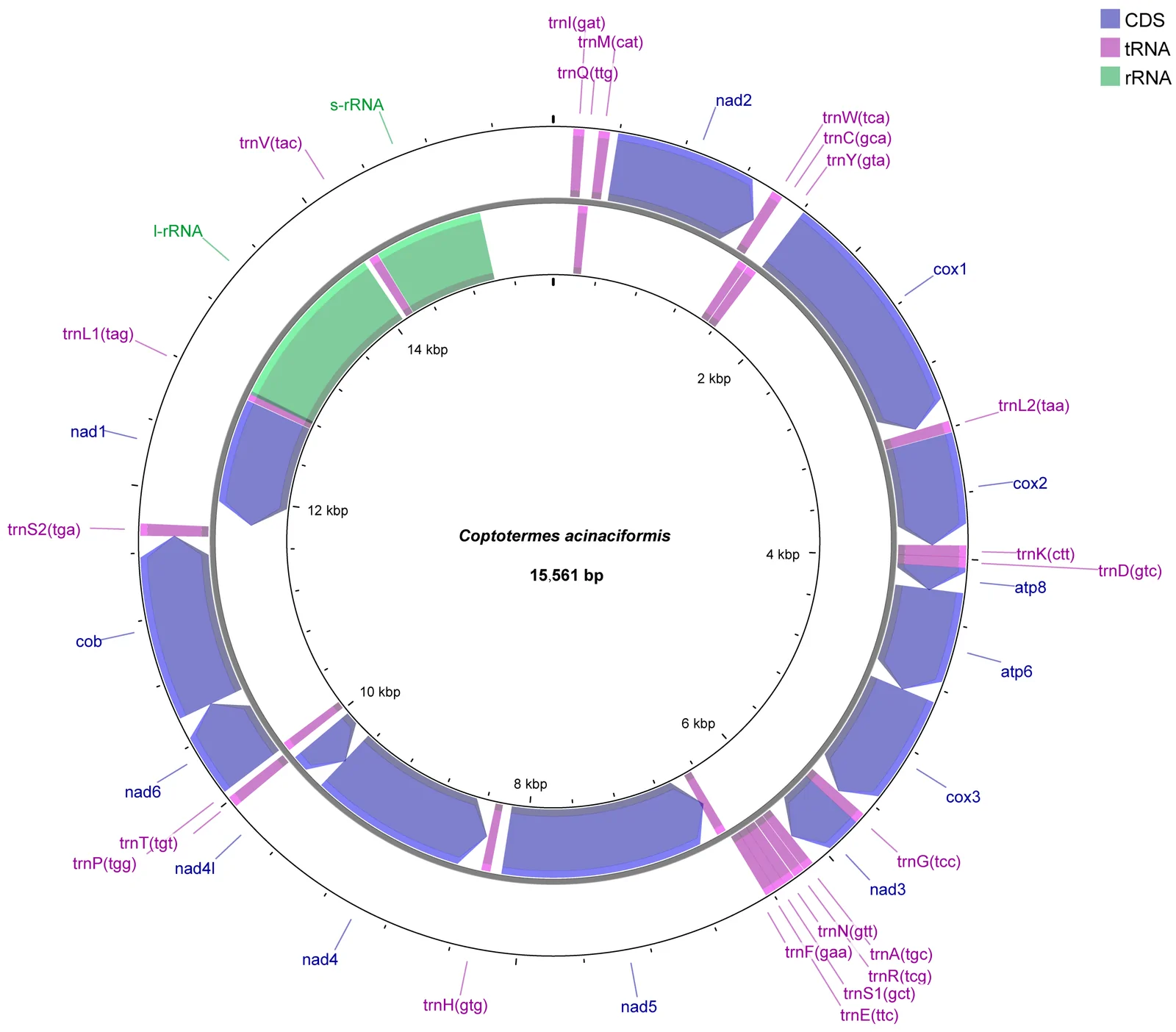
Circular mitochondrial genome map of *C. acinaciformis*. Gene abbreviations follow MITOS nomenclature.

Genome annotation identified the canonical set of 37 insect mitochondrial genes across all ten newly sequenced mitogenomes, comprising 13 protein-coding genes (PCGs), 22 transfer RNA genes (tRNAs), and two ribosomal RNA genes (rRNAs). All ten mitogenomes retained the ancestral insect gene arrangement [35] (Figure 1). The mitogenomes exhibited a pronounced A+T bias, with overall A+T content ranging from 64.4% (*O. formosanus*) to 66.7% (*Tumulitermes* sp. AU_W3). Additionally, the majority strand consistently displayed a negative GC-skew across all species, varying between −0.334 (*M. barneyi*) and −0.277 (*Tumulitermes* sp. AU_W3).

**Figure 2:**
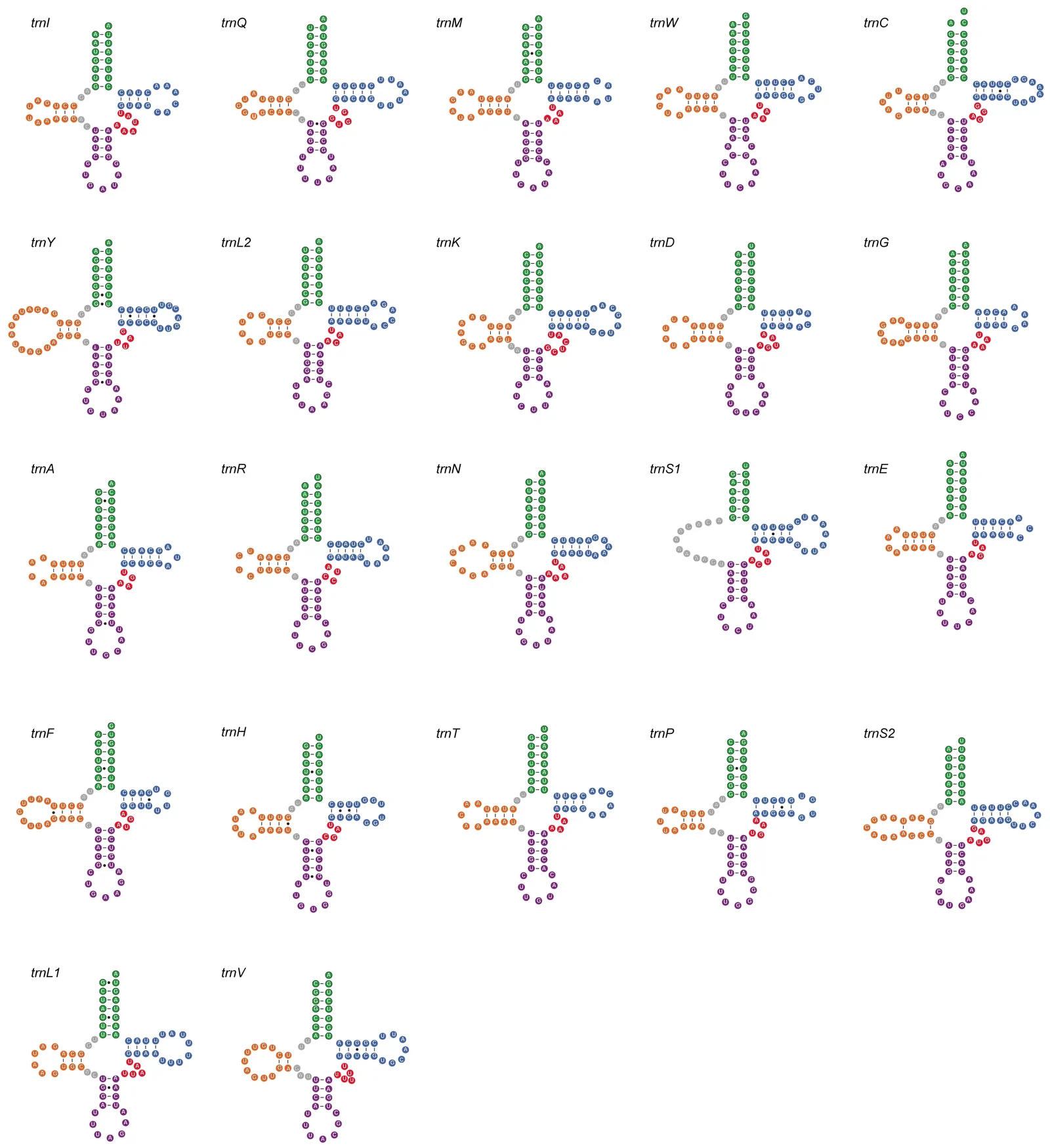
Predicted secondary structures of the 22 mitochondrial tRNA genes in *C. acinaciformis*. Colors indicate functional regions: green, aminoacyl acceptor arm; orange, DHU arm; purple, anticodon arm; red, variable loop or arm; and blue, TψC arm. Bars indicate Watson–Crick base pairs, whereas dots indicate G–U wobble pairs.

Individual tRNA lengths ranged from 62 bp (*trnF* in *R. qingjiangensis*, *R. leptomandibularis*, and *R. citrinus*) to 77 bp (*trnY* in *R. citrinus*, *R. kanmonensis*, *R. labralis*, *R. leptomandibularis*, and *R. qingjiangensis*). Although most tRNAs folded into the typical cloverleaf secondary structure (Figure 2), an atypical feature was observed in *trnS1(gct)*, which lacked the dihydrouridine (DHU) arm across all ten species. Overall, the tRNAs demonstrated high conservation in length and secondary structure among the newly sequenced species. Both rRNA genes (*l-rRNA* and *s-rRNA*) were encoded on the minority strand. The length of *l-rRNA* ranged from 1252 bp (*S. breinli*) to 1344 bp (*O. formosanus*), whereas *s-rRNA* varied from 811 bp (*C. acinaciformis* and *M.* barneyi) to 837 bp (*R. citrinus*, *R. leptomandibularis*, and *R. qingjiangensis*).

### 3.3. Phylogenetic Inference

The dataset included representatives of all 13 extant termite families, although the number of sampled species varied among families. All sampled families were recovered as monophyletic except Rhinotermitidae *sensu lato*, which was non-monophyletic under the revised classification. Although the inferred topologies differed among datasets, the major interfamily relationships were largely congruent. Although topologies varied across datasets, interfamilial relationships remained largely congruent. In the analyses based on the amino acid dataset (PCG_AA) and the two nucleotide datasets (PCG_NT123 and PCG_rRNA), *Mastotermes darwiniensis* (Mastotermitidae) was recovered as the sister group to all other termites. The four early-diverging families Hodotermopsidae, Stolotermitidae, Archotermopsidae, and Hodotermitidae formed two sister–group pairs: Hodotermopsidae + Stolotermitidae and Archotermopsidae + Hodotermitidae. The support values for these relationships are summarized in Table 1. Kalotermitidae was consistently recovered as an early-diverging lineage, although its precise relationship to the remaining major termite lineages should be interpreted in the context of the support values at the corresponding nodes.

**Figure 3:**
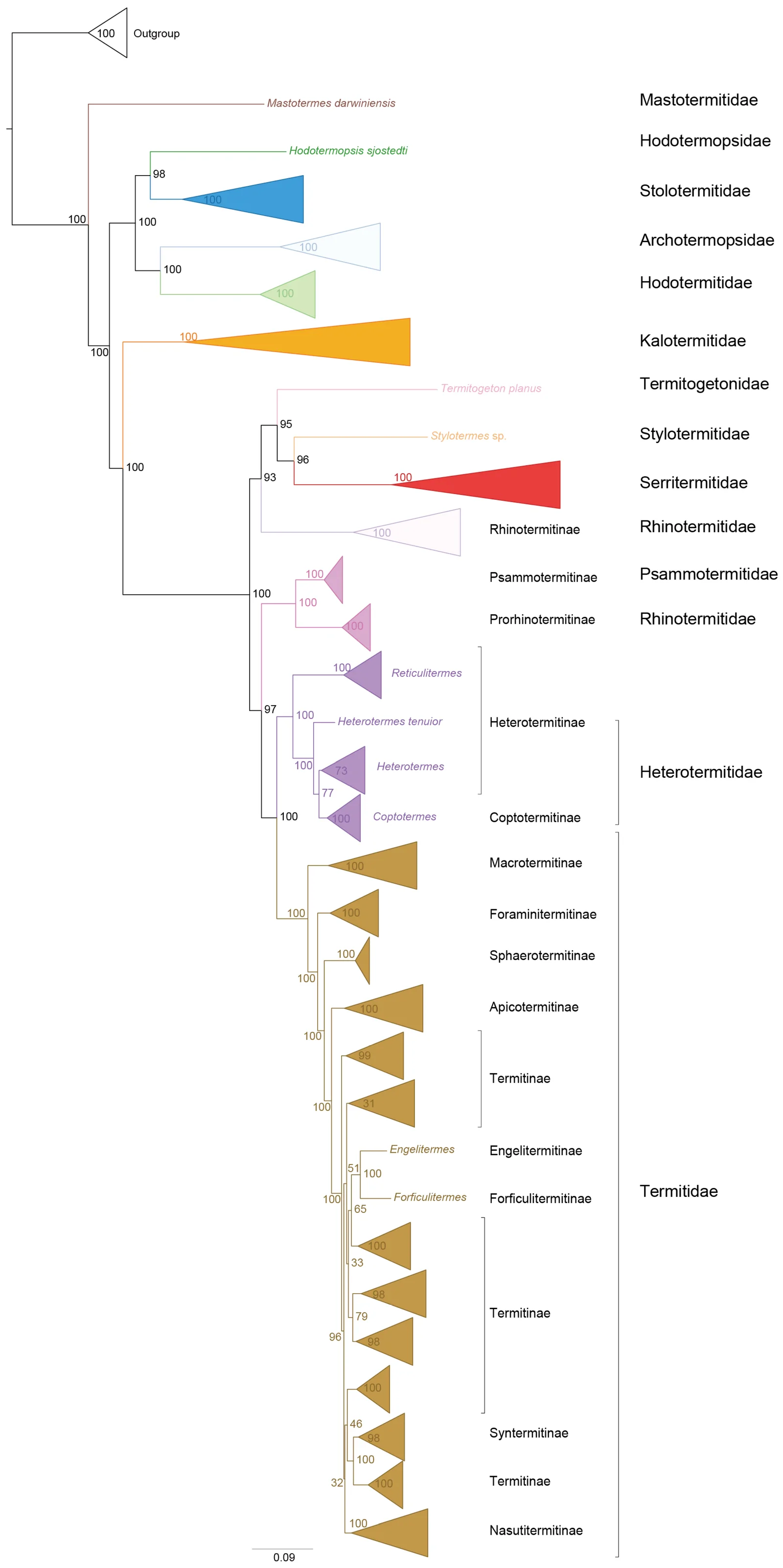
Phylogenetic trees inferred from the amino acid dataset PCG_AA using maximum likelihood analysis. Major lineages are collapsed for clarity, with triangle lengths corresponding to the longest terminal branch in each collapsed clade. Values at nodes represent ultrafast bootstrap support. The scale bar indicates substitutions per site. The full trees are provided in Supplementary Figure S2.

The principal topological differences involved the positions of Stylotermitidae, Termitogetonidae, and Serritermitidae, as well as their relationships to Rhinotermitidae *sensu stricto*. In the amino acid ML tree (PCG_AA; Figure 3 and Figure S2), Termitogetonidae, Stylotermitidae, and Serritermitidae formed a monophyletic clade. Within this clade, Stylotermitidae and Serritermitidae were recovered as sister groups (UFBoot = 96), which together formed a sister clade to Termitogetonidae (UFBoot = 95). This broader lineage was sister to Rhinotermitidae *sensu stricto* (UFBoot = 93), and together they formed the sister group to all remaining higher termites. In contrast, the nucleotide ML trees (PCG_NT123 and PCGrRNA, Figures S3 and S4) revealed a marked topological conflict: Stylotermitidae branched off independently as an early-diverging lineage (UFBoot = 100 in both datasets), whereas Termitogetonidae and Serritermitidae formed a strongly supported sister relationship (UFBoot = 91 in PCG_NT123; UFBoot = 100 in PCG_rRNA) positioned between Psammotermitidae and Rhinotermitidae *sensu stricto*. Additionally, the specific *Heterotermes* species recovered as sister to Coptotermitinae varied across phylogenetic analyses. In the amino acid ML tree (PCG_AA), all *Heterotermes* species except *Heterotermes tenuior* formed a sister clade to Coptotermitinae (UFBoot = 77). In contrast, nucleotide analyses recovered only a subset of *Heterotermes* species as sister to Coptotermitinae: three species (*H. tenuior*, *H. malabaricus*, and *H. vagus*) in the PCG_NT123 tree (UFBoot = 94), and two species (*H. malabaricus* and *H. vagus*) in the PCG_rRNA tree (UFBoot = 75). These differences indicate that the deeper relationships among these lineages were sensitive to the type of molecular data analyzed and do not support treating a single topology as definitively established.

Across both amino acid and nucleotide analyses, Psammotermitinae and Prorhinotermitinae were consistently recovered as sister groups. Additionally, Heterotermitidae was recovered as the sister group to monophyletic Termitidae. Within Heterotermitidae, Coptotermitinae was recovered as a monophyletic group, whereas Heterotermitinae was non-monophyletic. In the topology described below, *Heterotermes* was recovered as sister to Coptotermitinae, indicating that the traditional Heterotermitinae was non-monophyletic with respect to Coptotermitinae.

Within Termitidae, Macrotermitinae was sister to all other lineages. *Labritermes* and *Foraminitermes* were recovered as sister genera, forming the clade Foraminitermitinae. The two representatives of *Sphaerotermes* clustered into a distinct clade, supporting its removal from Macrotermitinae and elevation to the subfamily Sphaerotermitinae. *Engelitermes zambo* and *Forficulitermes planifrons* were recovered as sister taxa within an independent clade, despite their uncertain subfamily placements (Cubitermitinae/Termitinae and Forficulitermitinae/Termitinae, respectively). Apicotermitinae was monophyletic, whereas Termitinae was non-monophyletic, with its major lineages forming a grade. Finally, both Syntermitinae and Nasutitermitinae were recovered as monophyletic groups.

### 3.4. Compositional Heterogeneity and Substitution Saturation

AliGROOVE analyses of the amino acid dataset indicated no widespread compositional heterogeneity across the sampled taxa (Figure S5). Most taxa showed relatively low levels of compositional dissimilarity, although *Coatitermes aff. kartaboensis* (GenBank accession number PV938899) exhibited comparatively pronounced heterogeneity. Inspection of this sequence revealed that its aligned mitochondrial sequence was only 5154 bp long, resulting in a substantial amount of missing data. This incomplete sequence coverage may have contributed to the elevated heterogeneity signal. Despite this limitation, the species was placed with related taxa in the phylogenetic reconstruction. To evaluate the influence of potentially heterogeneous taxa on the inferred topology, we repeated the amino acid ML analysis after excluding *Coatitermes aff. kartaboensis* and *Neotermes luykxi*, the two taxa exhibiting comparatively high heterogeneity. The resulting tree showed no major topological changes relative to the original amino acid tree (Figure S6), suggesting that the overall topology was not strongly affected by the inclusion of these two taxa.

DAMBE saturation analyses indicated that the first and second codon positions showed no evidence of substantial substitutional saturation under either symmetrical- or asymmetrical-tree assumptions, with *Iss* values markedly lower than the corresponding critical values (Table S4). For the third codon position (32 OTUs), *Iss* (0.572) was lower than *Iss.cSym* (0.809) under the symmetrical-tree assumption but slightly exceeded *Iss.cAsym* (0.554) under the asymmetrical-tree assumption (*p* = 0.0260), suggesting that the data approached the saturation threshold under the latter assumption.

## 4. Discussion

With the rapid advancement of next-generation sequencing technologies, including both short-read and long-read sequencing, along with continuous updates to genome assembly and bioinformatic pipelines, obtaining, assembling, annotating, and analyzing insect mitochondrial genomes (mitogenomes) has become increasingly routine. In early molecular phylogenetic studies [15,16,17,25,46], mitogenomes played a crucial role in clarifying the higher-level relationships among termites, although some of these studies were constrained by relatively sparse taxon sampling. In the present study, we expanded the mitogenomic sampling of previous datasets to comprehensively test several recent hypotheses regarding termite classification, particularly at the family and subfamily levels. Specifically, we evaluate the monophyly and interrelationships of the various lineages within the current classification scheme.

Despite widespread consensus on the basal phylogenetic placement of Mastotermitidae, the precise relationships among other Termitoidea families, as well as several subfamilies within Rhinotermitidae and Termitidae, are not fully resolved [19].

### 4.1. Most Basal Clade: Mastotermitidae

*Mastotermes darwiniensis* Froggatt is the sole extant species of Mastotermitidae [2,69]. Mastotermitidae and the relict wood roaches (*Cryptocercus* spp. Scudder) share key biological traits including symbiotic hindgut protozoans for wood digestion, similar reproductive structures (such as oothecae), and subsocial behaviors, and serve as a critical evolutionary bridge between cockroaches and termites. The strong biological affinity between these taxa provides fundamental empirical support for the phylogenetic premise that termites represent a clade of eusocial cockroaches. Despite their distinct taxonomic status, both lineages exhibit notable transitional features across phylogenetic position, endosymbiotic microbiota, anatomical architecture, and ethological patterns. In our analyses, *Mastotermes darwiniensis* was consistently recovered as the most basal termite lineage and the sister group to all remaining Termitoidea. This result aligns with previous findings [7,17,18,19,20,22,23,25,27,28,29,41,45].

### 4.2. Basal Relationships Within Termitoidae

In the traditional seven-family classification system, basal termite lineages besides Mastotermitidae included Hodotermitidae and Termopsidae. Termopsidae historically comprised three subfamilies: Stolotermitinae; Termopsinae; and Porotermitinae. Termopsidae has since been confirmed to be non-monophyletic, leading to the elevation of its constituent subfamilies to family rank: Archotermopsidae includes the extant genera *Archotermopsis*, *Zootermopsis*, and *Hodotermopsis* [7]. Stolotermitidae (corresponding to Stolotermitinae) includes *Stolotermes* and *Porotermes* (formerly in Porotermitinae) [7]. Hodotermopsidae comprises only the extant genus *Hodotermopsis*, distributed from southern China and southern Japan to northern Vietnam [42,43]. Together, these four families constitute the group Teletisoptera [19,43]. Relationships among Hodotermitidae, Archotermopsidae, Stolotermitidae, and Hodotermopsidae, as well as their affinities relative to other termite lineages, have varied across previous studies. Using ultraconserved elements (UCEs), Hellemans et al. [20] recovered the topology Stolotermitidae + (Hodotermopsidae + Archotermopsidae), though their taxon sampling was sparse, with only one species per family. Wang et al. [43], using mitogenomes combined with nuclear ribosomal genes (5S, 5.8S, 18S, and 28S rRNA), recovered a sister–group relationship between Hodotermitidae and Archotermopsidae, with Stolotermitidae sister to (Hodotermopsidae + (Hodotermitidae + Archotermopsidae)). Conversely, a recent phylogenomic study by Hellemans et al. [19] placed Stolotermitidae as sister to the remaining three families, with Hodotermopsidae and Hodotermitidae forming a clade.

Across our three mitogenomic datasets, we recovered the topology (Hodotermopsidae + Stolotermitidae) + (Archotermopsidae + Hodotermitidae). This consistency suggests that the two constituent sister–group relationships are relatively stable within the present analyses. However, agreement among mitochondrial datasets does not independently validate deep-level relationships, as all datasets derive from the same linked organellar genome. Broader phylogenomic sampling, especially from independent nuclear loci, will be valuable for evaluating these relationships and determining whether the observed topology is robust to alternative data sources.

### 4.3. Kalotermitidae

In this study, Kalotermitidae was recovered as a monophyletic group and consistently placed as the sister group to Neoisoptera—the clade comprising the rhinotermitids (including Stylotermitidae and Serritermitidae) and Termitidae. This topology is consistent with previous findings [7,19,20,22,41]. Within Kalotermitidae, all genera represented by multiple species were monophyletic, with the exception of *Incisitermes*. Generic interrelationships within the family were broadly congruent with the findings of Buček et al. [70].

### 4.4. Rhinotermitidae

Historically, the family Rhinotermitidae Froggatt, 1897 [32] comprised seven subfamilies: Coptotermitinae, Heterotermitinae, Prorhinotermitinae, Psammotermitinae, Stylotermitinae, Termitogetoninae, and Rhinotermitinae. However, both morphological evidence [7] and molecular phylogenetic data [20,22,25,41,45] have consistently demonstrated the non-monophyly of the group. To address this issue, Hellemans et al. [19] recently proposed reclassifying these transitional lineages into four family-level groups by elevating the monogeneric subfamilies Prorhinotermitinae and Psammotermitinae to family status under Psammotermitidae, elevating Termitogetoninae to Termitogetonidae, unifying the paraphyletic Heterotermitinae and monophyletic Coptotermitinae into Heterotermitidae, and restricting Rhinotermitidae *sensu stricto* to the five genera within Rhinotermitinae (*Acorhinotermes*, *Dolichorhinotermes*, *Parrhinotermes*, *Rhinotermes*, and *Schedorhinotermes*).

Our mitogenomic analyses provide partial and qualified support for the taxonomic revisions proposed by Hellemans et al. [19]. Specifically, the five Rhinotermitinae genera (*Acorhinotermes*, *Dolichorhinotermes*, *Parrhinotermes*, *Rhinotermes*, and *Schedorhinotermes*) clustered together in a distinct clade that diverged earliest relative to other rhinotermitid lineages. Psammotermitinae and Prorhinotermitinae were also recovered as sister groups. In addition, Heterotermitinae and Coptotermitinae formed a clade corresponding to Heterotermitidae, which was recovered as sister to Termitidae. These results are broadly consistent with the revised classification proposed by Hellemans et al. [19], which recognizes Heterotermitidae as a separate family encompassing both Heterotermitinae and Coptotermitinae. Hellemans et al. [19] also recovered a subset of *Heterotermes* species (i.e., *Heterotermes vagus* and *Heterotermes tenuior*) as sister to Coptotermitinae, rendering Heterotermitinae paraphyletic.

However, the positions of Stylotermitidae, Termitogetonidae, and Serritermitidae varied between amino acid and nucleotide analyses. In the PCG_AA tree, Stylotermitidae and Termitogetonidae occurred in a clade associated with Serritermitidae, whereas the nucleotide trees placed Stylotermitidae as a separate early-diverging lineage and recovered Termitogetonidae and Serritermitidae elsewhere in the tree. Thus, although the analyses support the distinctiveness of several lineages formerly assigned to Rhinotermitidae, they do not consistently resolve the relationships among all these lineages. The position of Stylotermitidae, in particular, remains uncertain. These discrepancies may reflect differences in phylogenetic signal among amino acid and nucleotide datasets.

### 4.5. Termitidae

Termitidae has been consistently recovered as monophyletic across numerous studies [7,18,19,20,22,23,25,27,29,41,45], and our mitogenomic analyses recovered the same pattern. Within Termitidae, Macrotermitinae was recovered as the sister group to all remaining higher termites, congruent with several previous studies [15,17,22], but differing from other phylogenetic hypotheses [19,20,25,28]. Based on seven gene fragments, Legendre et al. [25] recovered *Sphaerotermes sphaerothorax* as the earliest-diverging lineage within Termitidae and classified it within Macrotermitinae. In contrast, transcriptome-based [28] and UCE-based phylogenies [19,20] placed *Sphaerotermes sphaerothorax* (as a distinct subfamily, Sphaerotermitinae) as sister to a monophyletic Macrotermitinae. Our analyses recovered the *Sphaerotermes* species as a distinct clade within Termitidae, consistent with its recognition as Sphaerotermitinae [15].

### 4.6. Limitations of Mitogenomic Phylogenetic Inference

Although mitogenomic data provide substantial information for termite phylogeny, several limitations warrant consideration. The three matrices (PCG_AA, PCG_NT123, and PCG_rRNA) represent alternative treatments of linked mitochondrial data rather than independent genomic sources.

DAMBE analyses indicated no substantial saturation at the first and second codon positions, whereas the third position approached the saturation threshold under the asymmetrical-tree assumption. Excluding the third position failed to satisfactorily resolve deeper phylogenetic relationships, particularly the placement of Mastotermitidae (Figure S7). Instead, Mastotermitidae was recovered nested deep within the ingroup, appearing as sister to a major clade comprising Stylotermitidae, Termitogetonidae, Serritermitidae, Rhinotermitidae, Psammotermitidae, Heterotermitidae, and Termitidae. We therefore retained all three codon positions in the primary nucleotide analyses but interpret deep-level relationships cautiously.

These limitations may explain the conflicting placements of Stylotermitidae, Termitogetonidae, and Serritermitidae. Discrepancies between the amino acid and nucleotide trees likely reflect variation in phylogenetic signal, compositional bias, or substitution saturation, which our analyses cannot conclusively distinguish. Consequently, relationships consistent across the three matrices should be viewed as robust to the data treatments examined, rather than independently validated. Resolving these contentious relationships and assessing potential systematic biases will ultimately require independent nuclear loci.

## 5. Conclusions

In this study, we sequenced ten new termite mitogenomes, all of which exhibited similar genome sizes and organizational features. The canonical set of 37 insect mitochondrial genes was identified in each of the newly sequenced genomes, and all retained the ancestral insect gene order. By integrating these data with previously published sequences, we assembled a large mitogenomic dataset comprising 553 termite ingroup species and 7 *Cryptocercus* outgroup species. Phylogenetic analyses based on this dataset recovered the monophyly of most families and supported the family-level status of several groups recently elevated from subfamilies within the traditional Rhinotermitidae. Furthermore, the inferred genera or inter-subfamily relationships within Kalotermitidae and Termitidae were largely consistent with findings from recent molecular studies. Substitution saturation analyses suggested that the third codon position approached the saturation threshold under the asymmetrical-tree assumption. Overall, our results provide a broad mitogenomic framework for termite phylogeny, while underscoring that unresolved deep-level relationships will require expanded taxon sampling and independent nuclear phylogenomic data.

## Figures and Tables

**Table 1:** Summary of key phylogenetic relationships, topological consistency, and ultrafast bootstrap support across the three mitogenomic datasets.

Key Phylogenetic Relationship	PCG_AA	PCG_NT123	PCG_rRNA	Interpretation
Mastotermitidae as sister to all other termites	UFBoot: 100	UFBoot: 100	UFBoot: 100	Consistent across datasets
Hodotermopsidae + Stolotermitidae	UFBoot: 98	UFBoot: 100	UFBoot: 91	Consistent across datasets
Archotermopsidae + Hodotermitidae	UFBoot: 100	UFBoot: 100	UFBoot: 100	Consistent across datasets
Kalotermitidae and the remaining major termite lineages	UFBoot: 100	UFBoot: 89	UFBoot: 100	Consistent across datasets
Placement of Stylotermitidae	Associated with Termitogetonidae and Serritermitidae, UFBoot: 96	Separate early-diverging lineage, UFBoot: 100	Separate early-diverging lineage, UFBoot: 100	Topological conflict
Placement of Termitogetonidae	Associated with Stylotermitidae and Serritermitidae, UFBoot: 95	Sister to Serritermitidae, UFBoot: 91	Sister to Serritermitidae, UFBoot: 100	Topological conflict
Placement of Serritermitidae	Associated with Stylotermitidae and Termitogetonidae, UFBoot: 96	Sister to Termitogetonidae, UFBoot: 91	Sister to Termitogetonidae, UFBoot: 100	Topological conflict
Psammotermitinae + Prorhinotermitinae	UFBoot: 100	UFBoot: 100	UFBoot: 100	Consistent across datasets
Heterotermitidae as sister to Termitidae	UFBoot: 100	UFBoot: 100	UFBoot: 100	Consistent across datasets
Monophyly of Termitidae	UFBoot: 100	UFBoot: 100	UFBoot: 100	Consistent across datasets
Macrotermitinae being sister to all other Termitidae	UFBoot: 100	UFBoot: 100	UFBoot: 100	Consistent across datasets

UFBoot: ultrafast bootstrap support.

## Data Availability

The ten newly sequenced mitogenomes have been deposited in GenBank under accession numbers QB020495–QB020504.

## Supplementary Materials

The following supporting information can be downloaded at: https://www.mdpi.com/article/10.3390/genes17101310/s1, Figure S1: Coverage plots for all mitogenomes newly sequenced in this study. Figure S2: Maximum likelihood phylogenetic tree of termites inferred from the PCG_AA amino acid dataset using IQ-TREE. Numbers at nodes represent ultrafast bootstrap support values. Figure S3: Maximum likelihood phylogenetic tree of termites inferred from the PCG_NT123 nucleotide dataset using IQ-TREE. Numbers at nodes represent ultrafast bootstrap support values. Figure S4: Maximum likelihood phylogenetic tree of termites inferred from the PCG_rRNA nucleotide dataset using IQ-TREE. Numbers at nodes represent ultrafast bootstrap support values. Figure S5: AliGROOVE heat map of pairwise sequence comparisons for the PCG_AA dataset. Mean sequence divergence scores range from −1 (high heterogeneity, red) to +1 (high similarity, dark blue). Taxa exhibiting marked sequence heterogeneity are indicated by larger font sizes and arrows. Figure S6: Maximum likelihood phylogenetic tree of termites inferred from the amino acid dataset using IQ-TREE, excluding *Coatitermes aff. kartaboensis* and *Neotermes luykxi*, the two taxa with elevated sequence heterogeneity. Numbers at nodes represent ultrafast bootstrap support values. Figure S7: Maximum likelihood phylogenetic tree of termites inferred from the PCG_NT12 dataset (including only the first and second codon positions) using IQ-TREE. Numbers at nodes represent ultrafast bootstrap support values. Table S1: Species analyzed in this study, including taxonomic classifications, GenBank accession numbers, and associated references [71,72,73,74,75,76,77,78,79,80,81,82,83,84,85,86,87,88,89]. Table S2: Correspondence between the traditional and revised family-level classification of termites. Table S3: Gene partitioning schemes and optimal substitution models determined by ModelFinder. Table S4: Results of substitution saturation analysis.

## Abbreviations

The following abbreviations are used in this manuscript:

Mitogenome	Mitochondrial genome
PCGs	Protein-coding genes
tRNAs	Transfer RNAs
DNA	Deoxyribonucleic acid
BOLD	Barcode of Life Data System
ID%	Identity percentage
bp	Base pair
ML	Maximum likelihood
UFBoot	Ultrafast bootstrap
Iss	Index of Substitution Saturation
Iss.cSym	Critical Index of Substitution Saturation assuming Symmetric Tree
Iss.cAsym	Critical Index of Substitution Saturation assuming Asymmetrical Tree
N_OTU_	Number of Operational Taxonomic Units
