# Supplementary material for: Phylogeny of Termites Inferred from Mitogenomic Data with a Large Taxon Sampling
Source: Genes (Basel). 2026 Oct 21;17(10):1310. doi: 10.3390/genes17101310 (PMC13704758; doi:10.3390/genes17101310)
Supplement: Supplementary file 1 [file genes-17-01310-s001.zip › Figure S2 PCG_AA_ML.pdf]

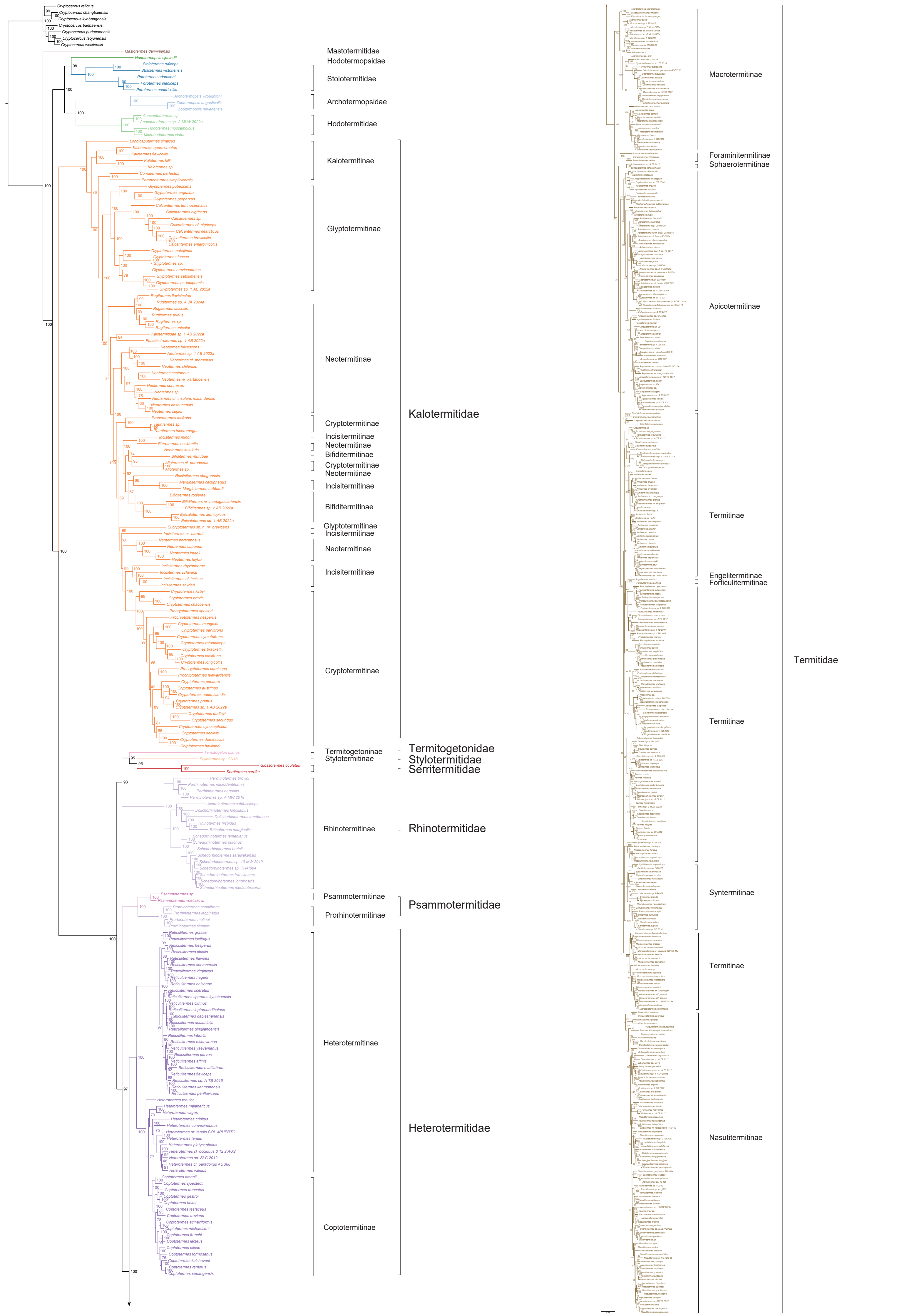

Mastotermitidae
Hodotermopsidae
Stolotermitidae
Archotermopsidae
Hodotermitidae
Kalotermitinae
Glyptotermitinae
Neotermitinae
Cryptotermitinae
Incisitermitinae
Neotermitinae
Bifiditermitinae
Cryptotermitinae
Neotermitinae
Incisitermitinae
Bifiditermitinae
Glyptotermitinae
Incisitermitinae
Neotermitinae
Incisitermitinae
Cryptotermitinae
Kalotermitidae
Termitogetoninae
Stylotermitinae
Termitogetonidae
Stylotermitidae
Serritermitidae
Rhinotermitinae
Rhinotermitidae
Psammotermitinae
Prorhinotermitinae
Psammotermitidae
Heterotermitinae
Coptotermitinae
Heterotermitidae
Macrotermitinae
Foraminitermitinae
Sphaerotermitinae
Apicotermitinae
Termitinae
Engelitermitinae
Forficulitermitinae
Termitinae
Syntermitinae
Termitinae
Nasutitermitinae
Termitidae
