# Supplementary material for: Phylogeny of Termites Inferred from Mitogenomic Data with a Large Taxon Sampling
Source: Genes (Basel). 2026 Oct 21;17(10):1310. doi: 10.3390/genes17101310 (PMC13704758; doi:10.3390/genes17101310)
Supplement: Supplementary file 1 [file genes-17-01310-s001.zip › Figure S3 PCG_NT123_ML.pdf]

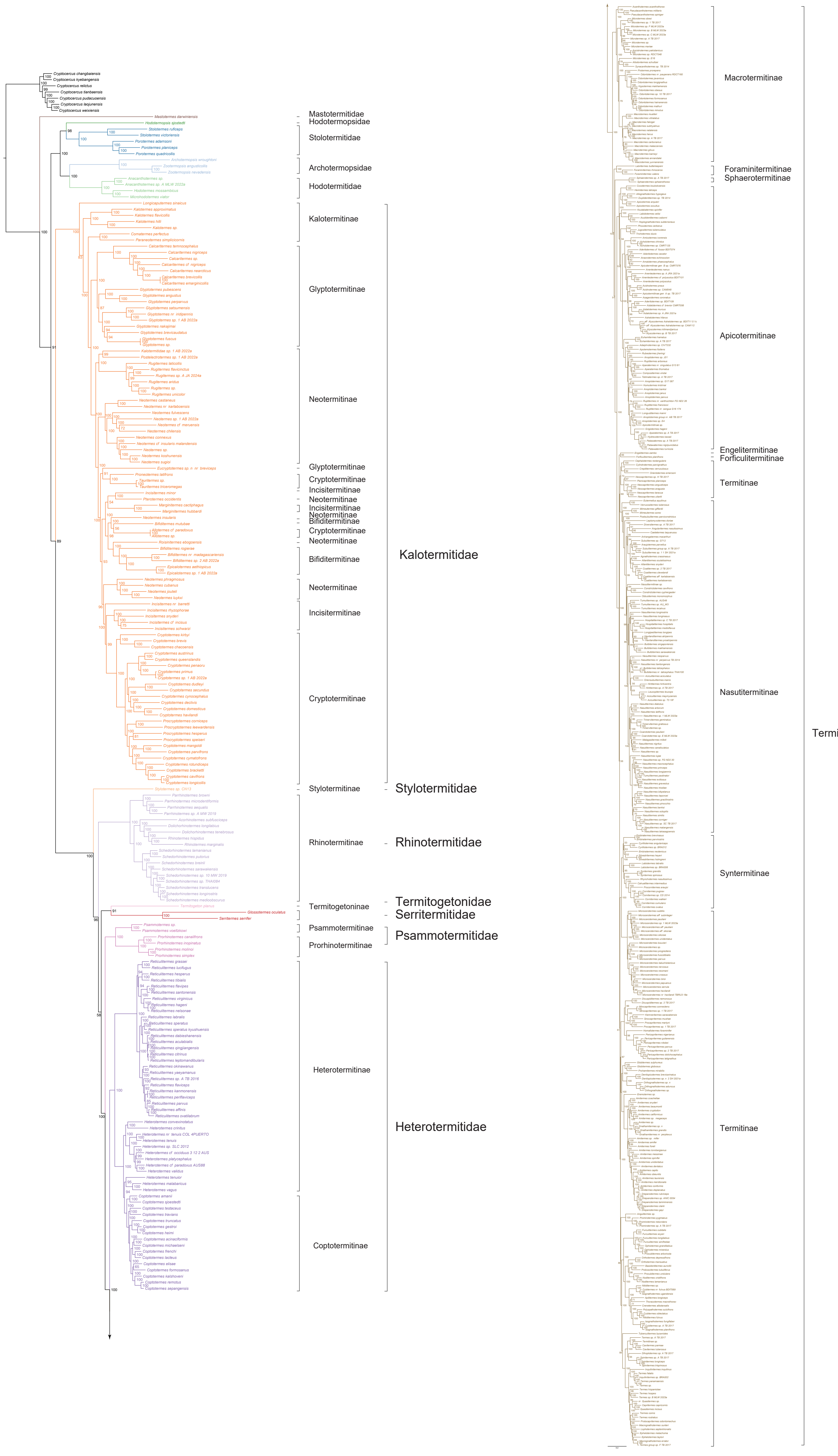

Mastotermitidae
Hodotermopsidae
Stolotermitinae
Archotermopsidae
Hodotermitidae
Kalotermitinae
Glyptotermitinae
Neotermitinae
Glyptotermitinae
Cryptotermitinae
Incisitermitinae
Neotermitinae
Incisitermitinae
Neotermitinae
Bifiditermitinae
Cryptotermitinae
Neotermitinae
Bifiditermitinae
Neotermitinae
Incisitermitinae
Cryptotermitinae
Kalotermitidae
Stylotermitinae
Stylotermitidae
Rhinotermitinae
Rhinotermitidae
Termitogetoninae
Termitogetonidae
Serritermitidae
Psammotermitinae
Psammotermitidae
Prorhinotermitinae
Heterotermitinae
Heterotermitidae
Coptotermitinae
Macrotermitinae
Foraminitermitinae
Sphaerotermitinae
Apicotermitinae
Engelitermitinae
Forficulitermitinae
Termitinae
Nasutitermitinae
Syntermitinae
Termitinae
Termitidae
