# Supplementary material for: Phylogeny of Termites Inferred from Mitogenomic Data with a Large Taxon Sampling
Source: Genes (Basel). 2026 Oct 21;17(10):1310. doi: 10.3390/genes17101310 (PMC13704758; doi:10.3390/genes17101310)
Supplement: Supplementary file 1 [file genes-17-01310-s001.zip › Figure S4 PCG_rRNA_ML.pdf]

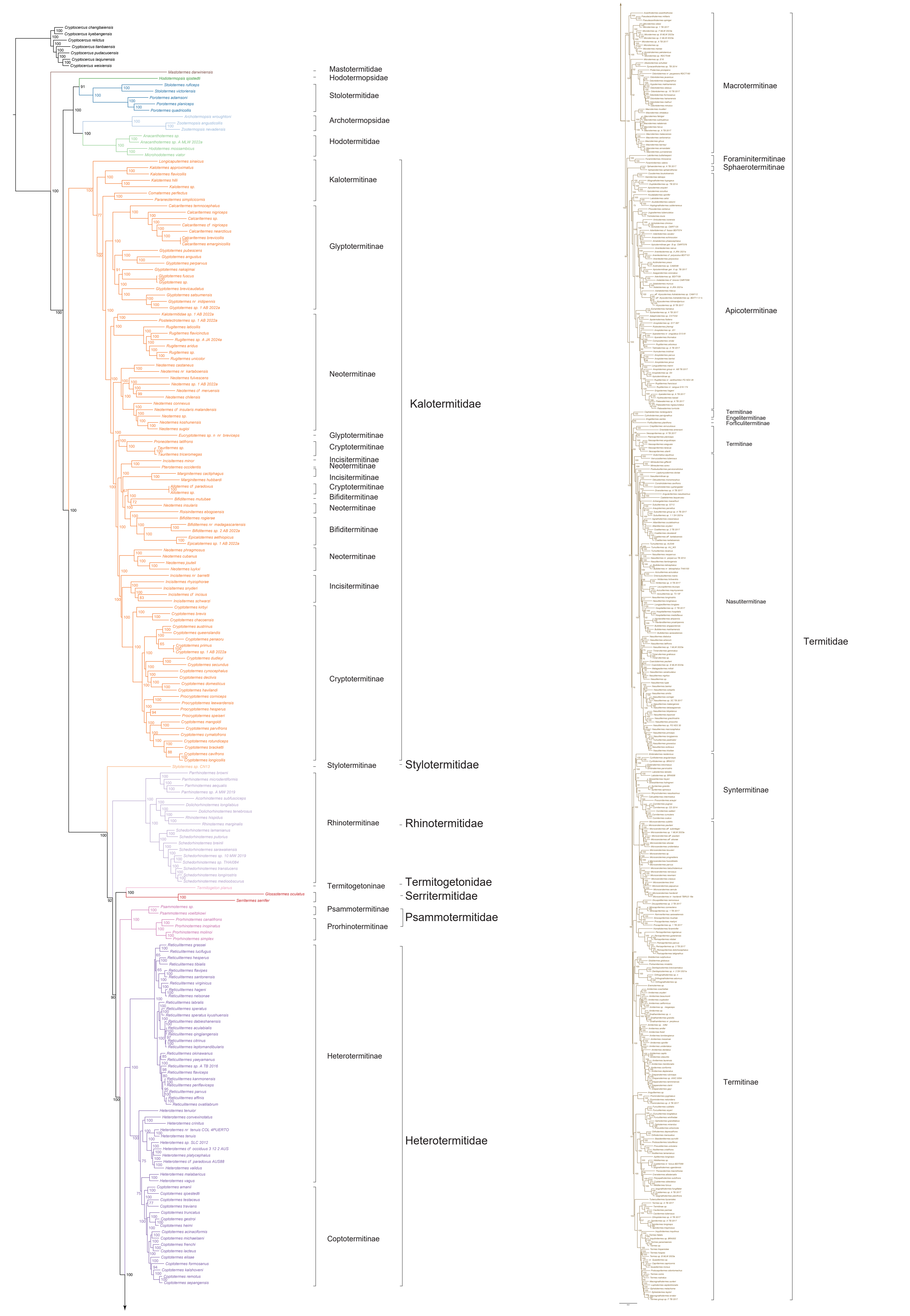

Cryptocercus changbaiensis
Cryptocercus kyebangensis
Cryptocercus relictus
Cryptocercus tianbaensis
Cryptocercus pudacuoensis
Cryptocercus laojunensis
Cryptocercus weixiensis
Mastotermes darwiniensis
Hodotermopsis sjostedti
Stolotermes ruficeps
Stolotermes victoriensis
Porotermes adamsoni
Porotermes planiceps
Porotermes quadricollis
Archotermopsis wroughtoni
Zootermopsis angusticollis
Zootermopsis nevadensis
Anacanthotermes sp.
Anacanthotermes sp. A MLW 2022a
Hodotermes mossambicus
Microhodotermes viator
Longicaputermes sinaicus
Kalotermes approximatus
Kalotermes flavicollis
Kalotermes hilli
Kalotermes sp.
Comatermes perfectus
Paraneotermes simplicicornis
Calcaritermes temnocephalus
Calcaritermes nigriceps
Calcaritermes sp.
Calcaritermes cf. nigriceps
Calcaritermes nearcticus
Calcaritermes brevicollis
Calcaritermes emarginicollis
Glyptotermes pubescens
Glyptotermes angustus
Glyptotermes perparvus
Glyptotermes nakajimai
Glyptotermes fuscus
Glyptotermes sp.
Glyptotermes brevicaudatus
Glyptotermes satsumensis
Glyptotermes nr. iridipennis
Glyptotermes sp. 1 AB 2022a
Kalotermitidae sp. 1 AB 2022a
Postelectrotermes sp. 1 AB 2022a
Rugitermes laticollis
Rugitermes flavicinctus
Rugitermes sp. A JA 2024a
Rugitermes aridus
Rugitermes sp.
Rugitermes unicolor
Neotermes castaneus
Neotermes nr. kartaboensis
Neotermes fulvescens
Neotermes sp. 1 AB 2022a
Neotermes cf. meruensis
Neotermes chilensis
Neotermes connexus
Neotermes cf. insularis malandensis
Neotermes sp.
Neotermes koshunensis
Neotermes sugioi
Eucryptotermes sp. n. nr. breviceps
Proneotermes latifrons
Tauritermes sp.
Tauritermes triceromegas
Incisitermes minor
Pterotermes occidentis
Marginitermes cactiphagus
Marginitermes hubbardi
Allotermes cf. paradoxus
Allotermes sp.
Bifiditermes mutubae
Neotermes insularis
Roisinitermes ebogoensis
Bifiditermes rogierae
Bifiditermes nr. madagascariensis
Bifiditermes sp. 2 AB 2022a
Epicalotermes aethiopicus
Epicalotermes sp. 1 AB 2022a
Neotermes phragmosus
Neotermes cubanus
Neotermes jouteli
Neotermes luykxi
Incisitermes nr. barretti
Incisitermes rhyzophorae
Incisitermes snyderi
Incisitermes cf. incisus
Incisitermes schwarzi
Cryptotermes kirbyi
Cryptotermes brevis
Cryptotermes chacoensis
Cryptotermes austrinus
Cryptotermes queenslandis
Cryptotermes penaoru
Cryptotermes primus
Cryptotermes sp. 1 AB 2022a
Cryptotermes dudleyi
Cryptotermes secundus
Cryptotermes cynocephalus
Cryptotermes declivis
Cryptotermes domesticus
Cryptotermes havilandi
Procryptotermes corniceps
Procryptotermes leewardensis
Procryptotermes hesperus
Procryptotermes speiseri
Cryptotermes mangoldi
Cryptotermes parvifrons
Cryptotermes cymatofrons
Cryptotermes rotundiceps
Cryptotermes bracketti
Cryptotermes cavifrons
Cryptotermes longicollis
Stylotermes sp. CN13
Parrhinotermes browni
Parrhinotermes microdentiformis
Parrhinotermes aequalis
Parrhinotermes sp. A MW 2019
Acorhinotermes subfusciceps
Dolichorhinotermes longilabius
Dolichorhinotermes tenebrosus
Rhinotermes hispidus
Rhinotermes marginalis
Schedorhinotermes lamanianus
Schedorhinotermes putorius
Schedorhinotermes breinli
Schedorhinotermes sarawakensis
Schedorhinotermes sp. 10 MW 2019
Schedorhinotermes sp. THAI084
Schedorhinotermes translucens
Schedorhinotermes longirostris
Schedorhinotermes medioobscurus
Termitogeton planus
Glossotermes oculatus
Serritermes serrifer
Psammotermes sp.
Psammotermes voeltzkowi
Prorhinotermes canalifrons
Prorhinotermes inopinatus
Prorhinotermes molinoi
Prorhinotermes simplex
Reticulitermes grassei
Reticulitermes lucifugus
Reticulitermes hesperus
Reticulitermes tibialis
Reticulitermes flavipes
Reticulitermes santonensis
Reticulitermes virginicus
Reticulitermes hageni
Reticulitermes nelsonae
Reticulitermes labralis
Reticulitermes speratus
Reticulitermes speratus kyushuensis
Reticulitermes dabieshanensis
Reticulitermes aculabialis
Reticulitermes qingjiangensis
Reticulitermes citrinus
Reticulitermes leptomandibularis
Reticulitermes okinawanus
Reticulitermes yaeyamanus
Reticulitermes sp. A TB 2016
Reticulitermes flaviceps
Reticulitermes kanmonensis
Reticulitermes periflaviceps
Reticulitermes parvus
Reticulitermes affinis
Reticulitermes ovatilabrum
Heterotermes tenuior
Heterotermes convexinotatus
Heterotermes crinitus
Heterotermes nr. tenuis COL 4PUERTO
Heterotermes tenuis
Heterotermes sp. SLC 2012
Heterotermes cf. occiduus 3 12 2 AUS
Heterotermes platycephalus
Heterotermes cf. paradoxus AUS88
Heterotermes validus
Heterotermes malabaricus
Heterotermes vagus
Coptotermes amanii
Coptotermes sjoestedti
Coptotermes testaceus
Coptotermes travians
Coptotermes truncatus
Coptotermes gestroi
Coptotermes heimi
Coptotermes acinaciformis
Coptotermes michaelseni
Coptotermes frenchi
Coptotermes lacteus
Coptotermes elisae
Coptotermes formosanus
Coptotermes kalshoveni
Coptotermes remotus
Coptotermes sepangensis
Mastotermitidae
Hodotermopsidae
Stolotermitidae
Archotermopsidae
Hodotermitidae
Kalotermitinae
Glyptotermitinae
Neotermitinae
Kalotermitidae
Glyptotermitinae
Cryptotermitinae
Incisitermitinae
Neotermitinae
Incisitermitinae
Cryptotermitinae
Bifiditermitinae
Neotermitinae
Bifiditermitinae
Neotermitinae
Incisitermitinae
Cryptotermitinae
Stylotermitinae
Stylotermitidae
Rhinotermitinae
Rhinotermitidae
Termitogetoninae
Termitogetonidae
Serritermitidae
Psammotermitinae
Psammotermitidae
Prorhinotermitinae
Heterotermitinae
Heterotermitidae
Coptotermitinae
Macrotermitinae
Foraminitermitinae
Sphaerotermitinae
Apicotermitinae
Termitinae
Engelitermitinae
Forficulitermitinae
Termitinae
Nasutitermitinae
Termitidae
Syntermitinae
Termitinae
