# Supplementary material for: Phylogeny of Termites Inferred from Mitogenomic Data with a Large Taxon Sampling
Source: Genes (Basel). 2026 Oct 21;17(10):1310. doi: 10.3390/genes17101310 (PMC13704758; doi:10.3390/genes17101310)
Supplement: Supplementary file 1 [file genes-17-01310-s001.zip › Figure S6 PCG_NT12_ML.pdf]

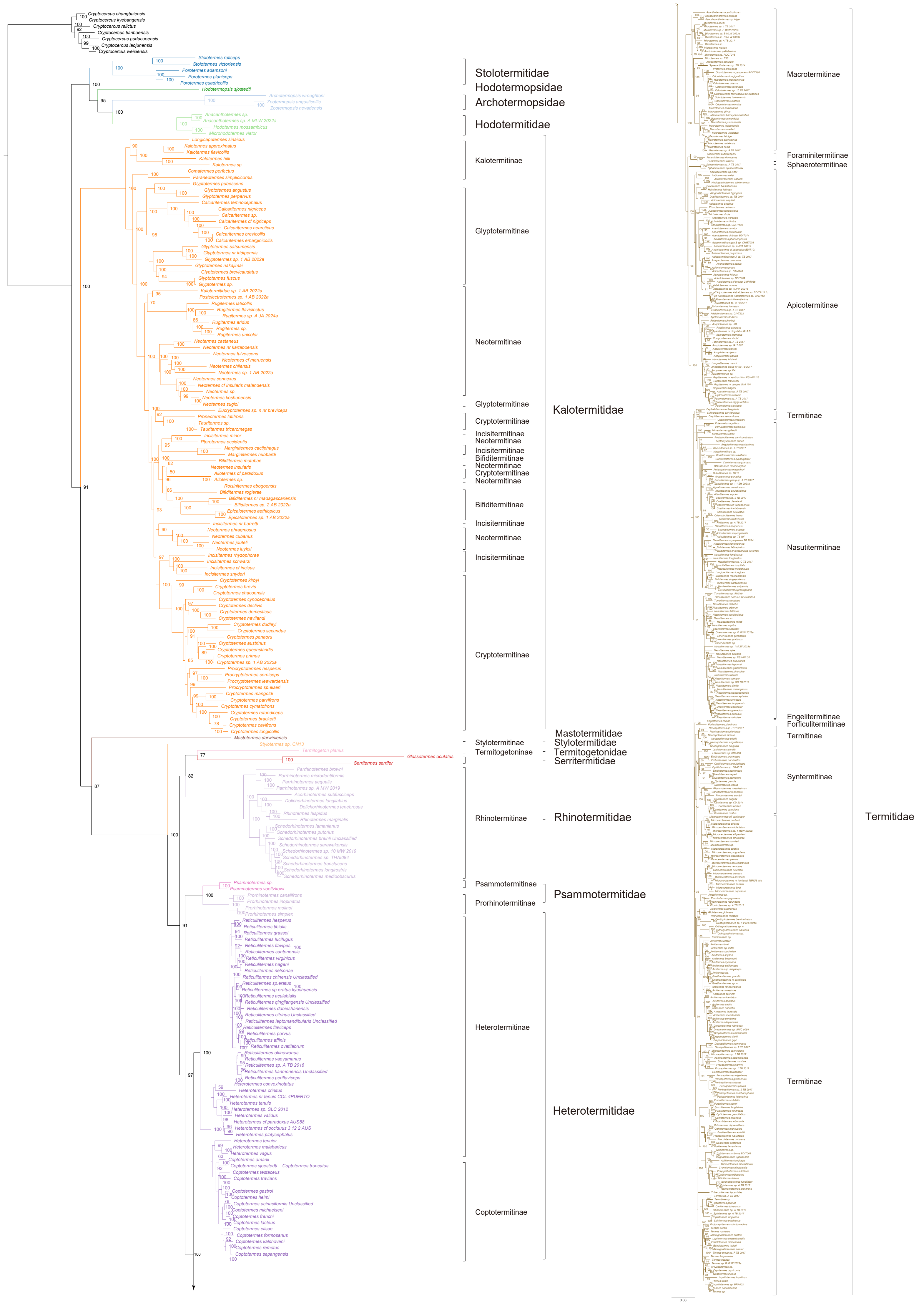

Stolotermitidae
Hodotermopsidae
Archotermopsidae
Hodotermitidae
Kalotermitinae
Glyptotermitinae
Neotermitinae
Glyptotermitinae
Cryptotermitinae
Incisitermitinae
Neotermitinae
Incisitermitinae
Bifiditermitinae
Neotermitinae
Cryptotermitinae
Neotermitinae
Bifiditermitinae
Incisitermitinae
Neotermitinae
Incisitermitinae
Cryptotermitinae
Kalotermitidae
Mastotermitidae
Stylotermitinae
Stylotermitidae
Termitogetoninae
Termitogetonidae
Serritermitidae
Rhinotermitinae
Rhinotermitidae
Psammotermitinae
Prorhinotermitinae
Psammotermitidae
Heterotermitinae
Heterotermitidae
Coptotermitinae
Macrotermitinae
Foraminitermitinae
Sphaerotermitinae
Apicotermitinae
Termitinae
Nasutitermitinae
Engelitermitinae
Forficulitermitinae
Termitinae
Syntermitinae
Termitinae
Termitidae
0.08
